# A Relationship between Reduced Nucleus Accumbens Shell and Enhanced Lateral Hypothalamic Orexin Neuronal Activation in Long-Term Fructose Bingeing Behavior

**DOI:** 10.1371/journal.pone.0095019

**Published:** 2014-04-15

**Authors:** Jacki M. Rorabaugh, Jennifer M. Stratford, Nancy R. Zahniser

**Affiliations:** 1 Department of Pharmacology, School of Medicine, University of Colorado Anschutz Medical Campus, Aurora, Colorado, United States of America; 2 Rocky Mountain Taste and Smell Center, Department of Cell and Developmental Biology, School of Medicine, University of Colorado Anschutz Medical Campus, Aurora, Colorado, United States of America; University of Chicago, United States of America

## Abstract

Fructose accounts for 10% of daily calories in the American diet. Fructose, but not glucose, given intracerebroventricularly stimulates homeostatic feeding mechanisms within the hypothalamus; however, little is known about how fructose affects hedonic feeding centers. Repeated ingestion of sucrose, a disaccharide of fructose and glucose, increases neuronal activity in hedonic centers, the nucleus accumbens (NAc) shell and core, but not the hypothalamus. Rats given glucose in the intermittent access model (IAM) display signatures of hedonic feeding including bingeing and altered DA receptor (R) numbers within the NAc. Here we examined whether substituting fructose for glucose in this IAM produces bingeing behavior, alters DA Rs and activates hedonic and homeostatic feeding centers. Following long-term (21-day) exposure to the IAM, rats given 8–12% fructose solutions displayed fructose bingeing but unaltered DA D1R or D2R number. Fructose bingeing rats, as compared to chow bingeing controls, exhibited reduced NAc shell neuron activation, as determined by c-Fos-immunoreactivity (Fos-IR). This activation was negatively correlated with orexin (Orx) neuron activation in the lateral hypothalamus/perifornical area (LH/PeF), a brain region linking homeostatic to hedonic feeding centers. Following short-term (2-day) access to the IAM, rats exhibited bingeing but unchanged Fos-IR, suggesting only long-term fructose bingeing increases Orx release. In long-term fructose bingeing rats, pretreatment with the Ox1R antagonist SB-334867 (30 mg/kg; i.p.) equally reduced fructose bingeing and chow intake, resulting in a 50% reduction in calories. Similarly, in control rats, SB-334867 reduced chow/caloric intake by 60%. Thus, in the IAM, Ox1Rs appear to regulate feeding based on caloric content rather than palatability. Overall, our results, in combination with the literature, suggest individual monosaccharides activate distinct neuronal circuits to promote feeding behavior. Specifically, long-term fructose bingeing activates a hyperphagic circuit composed in part of NAc shell and LH/PeF Orx neurons.

## Introduction

Feeding is a complex behavior driven by homeostatic (calorie-driven) and hedonic (pleasure-driven) mechanisms. Americans consume sugar in excess of homeostatic need, over 700 kcal per day, four times the recommended amount [1], [2]. Fructose and glucose are the major components of both commonly added sugars: high fructose corn syrup (55% fructose, 42% glucose and 3% polycose) and sucrose (a disaccharide of fructose and glucose). Fructose intake alone accounts for 10% of daily caloric intake, with over half of these calories coming from non-alcoholic beverages like sodas, sports drinks and fruit juices [1]. Despite being found together in many foods, fructose and glucose have distinct metabolic reinforcing profiles, which influence peripheral and central aspects of food intake and reward (reviewed here [3], [4]). Briefly, compared to glucose, fructose metabolism is poorly regulated; and the first step in fructose metabolism depletes cellular ATP levels by the rapid phosphorylation of fructose [5]. Within the hypothalamus, fructose-induced decreases in the ATP to AMP ratio stimulate feeding by activating the AMP kinase/malonyl-CoA signaling system, the same mechanism that glucose inhibits to produce satiety [5], [6]. Glucose, compared to fructose, ingestion produces a greater reduction in cerebral blow flow within the hypothalamus and enhances the functional connectivity of the hypothalamus to the striatum, a hedonic feeding center [7]. Thus, while it is clear that fructose directly interacts with homeostatic feeding mechanisms in the hypothalamus, less is known about the interactions of fructose with hedonic feeding mechanisms, which converge within the nucleus accumbens (NAc).

Both repeated sucrose ingestion and acute intragastric glucose infusion have been shown to increase neuronal activation, as determined by c-Fos immunoreactivity (Fos-IR), in hedonic areas such as the central amygdala (CeA) and the NAc shell and core [8], [9]. Recent work has shown that either sucrose or glucose given in the intermittent access model (IAM) produces signatures of hedonic feeding including bingeing behavior that is accompanied by sustained dopamine (DA) release or altered DA receptors within the NAc shell, respectively [10]–[12]. In the IAM, rats are cycled between 12 hr of food deprivation and 12 hr concurrent *ad libitum* access to a sugar solution and chow. In order to encourage bingeing, food access begins 4 hr into the dark cycle, making the animals miss their first natural meal. Over the course of the IAM, rats develop sugar bingeing behavior, characterized by a large sugar meal within the 1^st^ hr of food presentation, while chow intake remains constant [10]. Fructose has never been investigated in the IAM; and its hedonic characteristics, including the role of DA receptors, are unknown. We reasoned that because the IAM synchronizes sugar intake into large binges, it would be a useful tool for investigating whether fructose ingestion alters neuronal activation in hedonic and homeostatic feeding centers.

The lateral hypothalamus (LH) is a complex brain region that influences feeding by integrating information about external stimuli (smell, taste, light/dark cycle) and internal stimuli (energetic need, reward, associated memories) and transmitting it to areas important for decision making, attention, pleasure and endocrine function [13]. The LH and perifornical area (PeF) contain orexin (or hypocretin; further referred to here as Orx) neurons, which provide an important connection between homeostatic and hedonic feeding centers. Orx neurons found in the dorsomedial hypothalamus (DMH) are important in arousal, energy expenditure and stabilizing sleep/wake cycles [14], [15]. In addition to receiving inputs from other homeostatic nuclei within the hypothalamus, Orx neurons are sensitive to circulating hormones and local glucose levels [16]–[18]. Among many other regions, Orx neurons project to reward structures including the ventral tegmental area (VTA), where mesolimbic DA neurons originate, and the NAc shell, a terminal region of mesolimbic DA neurons [19]–[22]. NAc shell neurons send GABAergic projections to the LH and ventral pallidum (VP) [23]–[25]. Although NAc shell medium spiny projection neurons do not directly innervate Orx neurons, they form a functional feeding circuit, potentially through glutamatergic VP projections to the LH [26], [27]. This circuit enables chemical inhibition of NAc shell neurons to activate LH/PeF Orx neurons through two pathways, resulting in short-lived hyperphagia [26]–[28]. Further, in hypothalamic slices Orx neuron firing is reduced when exposed to high glucose, but not fructose, levels [17]. *In vivo* this would result in decreased Orx release after a glucose- or starch-rich meal but sustained Orx release following fructose ingestion. These observations suggest that Orx could be a unique contributor to fructose intake by acting within the NAc shell feeding circuit.

Orx neurons release two Orx peptides, Orx-A (or hypocretin-1) and Orx-B (or hypocretin-2), cleaved from a single propeptide [14]. Orx-A binds to Orexin 1 and 2 receptors (Ox1Rs and Ox2Rs) with similar affinities whereas Orx-B binds preferentially to Ox2Rs [14], [29]. Both Ox1Rs and Ox2Rs are located in feeding- and arousal-related brain areas where they subserve different functions [30]–[33]. In freely fed rats, an intracerebroventricular (i.c.v.) injection of Orx-A stimulates feeding through Ox1R activation [14], [34]. Systemic antagonism of Ox1Rs with SB-334867 decreases palatable food self-administration without altering arousal [35]–[39]. Conversely, an i.c.v. injection of Orx-B inhibits high-fat feeding; and systemic Ox2R antagonism enhances the transition into sleep [40], [41]. Interestingly, VTA application of Orx-A and Orx-B increases extracellular DA levels in the NAc shell [42], [43]. In the NAc shell Ox1Rs and Ox2Rs are involved in Orx-A- induced feeding behavior and locomotor activity, respectively [44], [45]. Thus, Orx-A stimulation of Ox1Rs appears to play an important role in enhancing overall feeding and motivation to eat and, therefore, could stimulate fructose bingeing behavior.

Here we investigated whether fructose can produce bingeing in the IAM and, in turn, activate hedonic and homeostatic feeding centers. We specifically focused on Orx-A neurons and Ox1R signaling as they could specifically enhance fructose feeding. Additionally, we examined whether fructose bingeing, like glucose bingeing, alters NAc DA D1R and D2R number. Fructose represents a significant and increasingly larger portion of the American diet; yet, the neuronal circuits influencing fructose consumption remain understudied. Gaining a greater understanding of the homeostatic and hedonic pathways mediating fructose intake may provide insights about curbing sugar and palatable food intake in humans.

## Methods

### Ethics Statement

The University of Colorado Denver IACUC approved all procedures under protocol number 39511(07)1D. This research program operates in accordance with the National Institutes of Health’s and National Research Council’s guidelines (Guide for Care and Use of Animals, 8^th^ Edition, 2011).

### Animals

A total of 96 male outbred Sprague-Dawley rats (Charles Rivers Laboratories, Wilmington, MA) weighing 200–220 g on arrival were used for these experiments. Rats were singly housed with food (Teklad 2020X chow: 3.1 kcal/g, 24% protein, 16% fat, 60% carbohydrate; Harlan Laboratories, Denver, CO) and water available *ad libitum* for 1 week prior to commencing experiments. A phase-shifted 12-hr light-dark cycle was used throughout all experiments (lights on 0300–1500).

### Chemicals

Unless otherwise stated, all chemicals were purchased from Sigma-Aldrich Co. (St. Louis, MO).

### Intermittent Access Model (IAM)

**Figure 1:**
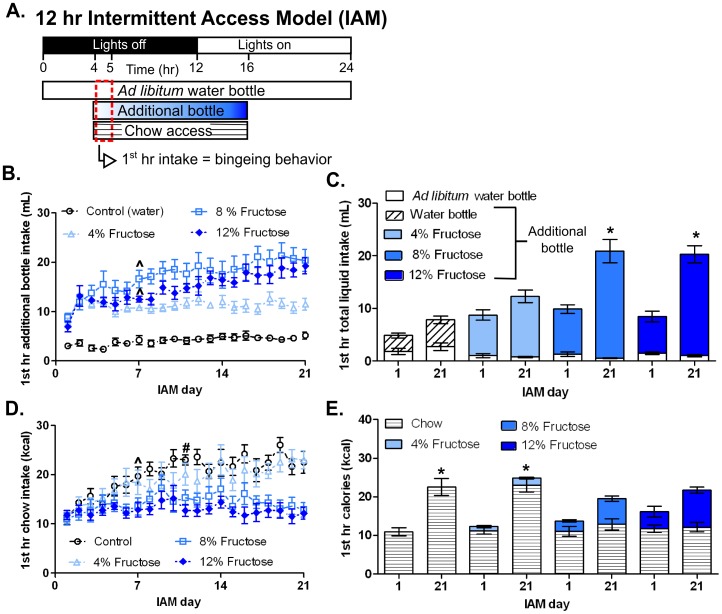
12-hr IAM produced concentration-dependent fructose bingeing. **A:** Schematic diagram showing the timeline of the daily light cycle and water, fructose and chow access during the IAM. Bingeing was defined as a statistically significant increase in the 1^st^ hr (dashed red box) fructose or chow intake between day 1 and subsequent diet days. **B:** Time course of 1^st^ hr additional bottle intake throughout IAM for the four treatment groups: control (a second novel water bottle), 4% fructose, 8% fructose and 12% fructose. ∧ Denotes the day at which stable fructose bingeing was achieved in the 8 and 12% fructose groups. **C:** 1^st^ hr total liquid intake of both the *ad libitum* water bottle (white columns) and the additional bottle that contained either water (diagonal striped column), or 4%, 8%, or 12% fructose solution (progressively darker blue columns) on the first and last day of the IAM. **D:** 1^st^ hr chow intake for the four groups throughout IAM. ∧, # Denotes the day at which stable chow bingeing was achieved in control and 4% fructose groups, respectively. **E:** 1^st^ hr caloric intake represented as chow (horizontal striped column) and fructose (blue column) calories on the first and last day of IAM. Mean values ± SEM (n = 8/group). *p<0.05 within group 1-way RMANOVA compared to day 1 with Tukey’s *post hoc* analysis.

The IAM of sugar bingeing was adapted from previously published studies with sucrose or glucose [10], [12]. On day 1 of IAM, 4 hr into the light cycle (0700 hr), all food was removed from the cages. The animals were re-fed 12 hr later (1900 hr) with chow and an additional bottle, which contained either water (control group) or a fructose solution (fructose group). All rats had 24-hr access to a water bottle, the *ad libitum* water bottle (Fig. 1A). Thus, during the 12-hr of food access, both the *ad libitum* water bottle and the additional bottle were present in the cage. The placement of the additional bottle was randomized to either the left or right side of the cage. Fructose was purchased from Fischer Scientific (Waltham, MA) and fructose solutions were fresh made daily. Fructose, chow and water intake were recorded for each rat following the 1^st^ hr and the entire 12-hr of food access. The 1^st^ hr of fructose or chow intake was used to determine bingeing behavior (see “Data Analysis”). Rats were weighed daily. IAM experiments were conducted using 4 cohorts referred to as: (#1) “fructose concentration-response (C-R)”, (#2) “long-term IAM”, (#3) “short-term IAM”, and (#4) “Ox1R antagonist”. For the fructose C-R experiment, the fructose groups were given either a 4%, 8% or 12% fructose solution. An 8% fructose solution was used for all other experiments. The IAM was maintained for 21 days (fructose C-R and long-term IAM), 2 days (short-term IAM) or 22 days (Ox1R antagonist). Group size was n = 8 for all experiments, except the Ox1R antagonist experiment wherein n = 6–9.

### Fat Pad Dissections

The epipdidymal and renal fat pads were removed from rats in the fructose C-R experiment. Following decapitation, an abdominal incision was made and fat pads were removed from atop both testis and kidneys, weighed and discarded.

### DA D1 and D2 Receptor Quantitative Autoradiography

Following day 21 of IAM, fructose C-R rats were decapitated, and the brains were flash frozen on dry ice. Using a cryostat, the brains were cut at the level of the NAc (+2.2 to +0.7 mm from bregma) into 15 µm sections, and two non-sequential sections were mounted onto gelatin-coated slides. Slides were randomized between the two radioligands and amongst the concentrations of radioligand and then processed for D1R and D2R quantitative autoradiography using direct saturation assays adapted from previously published study in the Zahniser lab [46]. Slides were thawed and preincubated in assay buffer (20 mM Hepes, 154 mM NaCl, and 0.1% bovine serum albumin (BSA); pH 7.4) for 20 min at 37°C. Total D1R receptor binding was assessed by incubating slides in assay buffer containing 6 concentrations (0.05–10 nM) of [^3^H] SCH-23390 (Perkin-Elmer) and 100 nM ketanserin for 60 min at 37°C. Nonspecific binding was assessed in the presence of 1 µM SCH-39166 for all concentration of radioligand. Slides were washed twice for 10 min each at 4°C in wash buffer (10 mM Tris HCl, 154 mM NaCl pH 7.4). For D2Rs total receptor binding was assessed by incubating slides in assay buffer containing 6 concentrations (10–600 pM) of [^3^H] spiperone (Perkin-Elmer, Waltham MA), and 100 nM ketanserin for 100 min at 37°C. Nonspecific binding was assessed in the presence of 200 µM S-sulpiride for all concentrations of radioligand. Slides were washed twice for 20 min at 4°C in wash buffer. Following the wash all slides were dipped in ice-cold water, blown dry with a gentle air stream and allowed to dry overnight. Slides were then exposed along with tritium standards (American Radiolabeled Chemicals, St. Louis, MO) to film (Kodak MR) for 9 wk for [^3^H] SCH-23390 or 13 wk for [^3^H] spiperone. All films were processed and areas of interest were outlined and analyzed using MCID image analysis software (MCID Image Analysis, UK). Saturation curves were fit to a single-site rectangular hyperbola model, and the number of receptors (Bmax) and receptor affinity (Kd) were derived using Prism (GraphPad Software, San Diego, CA).

### Immunohistochemistry

On the final day of the IAM, day 21 (long-term IAM), day 2 (short-term IAM), or day 22 (Ox1R antagonist pretreatment), rats were deeply anesthetized with urethane (1.5 mg/kg; i.p.) 90 min after the daily food presentation and injected with heparin (200 Units) at the apex of the heart. Using a Masterflex peristaltic pump (Vernon Hills, IL), rats were transcardially perfused first with 100 mL of ice-cold 0.05 M potassium phosphate buffered saline (pH 7.4; KPBS), followed by 175 mL of ice-cold 4% paraformaldehyde (PFA) in KPBS. The brains were removed and post-fixed in a solution containing a 1∶1 ratio of 4% PFA: 30% sucrose for 1 hr at 4°C and then equilibrated in 30% sucrose in KPBS. Brains were frozen, embedded in optical cutting temperature compound and cut into 30 µm coronal sections at the following distance from bregma: mPFC, +2.7 to +1.7 mm; NAc, +2.2 to 1.0 mm; and hypothalamus and amygdala, −1.8 to −3.6 mm, using a cryostat and then stored at −20°C in cryoprotectant.

All sections were washed four times for 10 min each in 0.1 M sodium PBS (pH 7.4; PBS) and blocked in PBS containing 2% normal donkey serum, 0.03% Triton-X, 1% BSA for 1 hr at room temperature. A 1∶10000 rabbit anti-c-Fos antibody (sc-52; Santa Cruz Biotech, Dallas TX) was added to the blocking buffer for all sections and allowed to incubate for 48 hr at 4°C. To the hypothalamic and amygdala sections an additional 1∶1500 goat anti-Orx-A antibody (sc-8070; Santa Cruz) was added. All sections were washed three times for 10 min each in PBS and incubated for 2 hr in blocking buffer plus 1∶500 donkey anti-rabbit Alexa488 (A21206; Life Tech, Grand Island, NY), and 1∶500 Neurotrace 640/660 (N21483; Life Tech), a deep red fluorescent Nissl stain. Hypothalamic sections were also incubated with 1∶500 donkey anti-goat Alexa 568 (A11057; Life Tech). All sections were washed three times for 10 min each in PBS, once for 10 min in 0.05 M phosphate buffer, mounted and cover-slipped with Fluormount (Southern Biotech, Birmingham, AL).

### Image Collection and Analysis

Whole slide images of brain sections were photographed using Surveyor by Objective Imaging software (Cambridge, UK; http://www.objectiveimaging.com/Surveyor/OI_Turboscan.htm) with a black and white Leica DFC 365FX camera on a Leica DM6000B microscope. For each fluorophore, a series of 10X images, aligned in a grid, were obtained using the Multiscan setting. Each channel (FITC, Texas Red and Cy-5) was obtained sequentially to prevent side-band excitation of the different fluorophores. Images were then stitched together in real time using the best focus algorithm in the Surveyor software, which yielded a mosaic image of the whole microscope slide. Then images of whole brain sections were exported as single images using the Surveyor selection tool. Images were analyzed using a self-written Matlab program (Matlab code available upon request). Brain regions of interest were outlined in reference to the rat brain atlas of Paxinos and Watson (2006) and included the NAc shell and core, medial prefrontal cortex (mPFC), basolateral amygdala (BLA), central amygdala (CeA), LH/PeF, dorsomedial hypothalamus (DMH), ventromedial hypothalamus (VMH), and paraventricular hypothalamus (PVN). To ensure double counting did not occur in anatomically adjacent brain regions, the borders of previously counted brain regions were displayed on the image prior to additional outlining. For each rat, 3–4 brain sections were analyzed per brain region; and a single mean value was determined for each rat that was then used to determine the group mean and SEM.

### Ox1R Antagonist

On day 22 of IAM, 30 min prior to food presentation (18∶30), control and fructose groups received an intraperitoneal (i.p.) injection of either vehicle (10% 2-hydroxpropyl β-cyclodextrin and 2% DMSO in sterile water) (n = 8 and n = 9, respectively) or a suspension of SB-334867, the Ox1R antagonist (30 mg/kg; generous gift of the NIDA Drug Supply Program, Bethesda, MD) in vehicle (n = 7 and n = 6, respectively). This SB-334867 dose and schedule has been shown to reduce chow intake as well as cocaine, ethanol and sucrose self-administration [35], [47]. Injections were made at 4 mL/kg-body weight.

### Data Analysis

Group data are expressed as mean value ± SEM, and significance was set at p<0.05. All statistical tests were performed using either Prism or SPSS (IBM, Armonk, NY) software. Bingeing behavior was defined as a significant increase in 1^st^ hr fructose or chow intake between day 1 and subsequent days of IAM. A within-group 1-way repeated measures analysis of variance (RMANOVA) was used to determine bingeing in all experiments, except for the short-term IAM where a paired t-test was used to determine bingeing. Comparisons across feeding groups over time or between drug treatments were made using a 2-way RMANOVA. A 1-way ANOVA was used to compare D1R and D2R Bmax and Kd values across feeding groups. Unpaired t-tests were used for comparisons of Fos-IR in the long and short-term IAM experiments. Within-diet 1-way ANOVAs were used to compare Fos-IR in the Ox1R antagonist experiment. Tukey’s *post hoc* analysis was used to compare groups following all significant ANOVAs.

## Results

### IAM Produced Fructose Bingeing without altering DA D1R or D2R Number

Initially, we examined whether fructose in the IAM would produce bingeing and alter D1R or D2R number. In the IAM, rats had 24-hr access to water but were cycled between 12 hr of food deprivation and 12 hr of *ad libitum* food access, beginning 4 hr into the dark cycle (Fig. 1A). During food access, groups were given chow and an additional bottle that contained water (control group), or a 4, 8 or 12% fructose solution (4%, 8% or 12% fructose group, respectively). Like previous IAM studies, we defined bingeing as a statistically significant increase in 1^st^ hr fructose or chow intake between day 1 and subsequent IAM days (Fig. 1A) [10] **.** By day 7 of the IAM, the 8% and 12% fructose groups showed stable bingeing behavior [8% fructose: F_(20,140)_ = 7.39, p<0.0001; 12% fructose: F_(20,140)_ = 8.74, p<0.0001; Fig. 1B]. However, these groups failed to meet chow bingeing criteria (Figs. 1D and 1E).

In contrast, the control animals and 4% fructose animals did not change liquid intake (Figs. 1B and 1C), but exhibited chow bingeing by days 7 and 11 of the IAM, respectively [Control: F_(20,140)_ = 6.72, p<0.0001; 4% fructose: F_(20,140)_ = 5.80, p<0.001; Fig. 1D]. Importantly, all the feeding groups consumed similar 1^st^ hr calories (Fig. 1E). The total (12-hr) intake of fructose and chow mirrored 1^st^ hr intake results. With one exception, by the end of the IAM the 8% and 12% fructose groups consumed more total calories than the 4% fructose and control groups [F_(3,28)_ = 8.95, p<0.01; data not shown]. However, this did not differentially affect weight gain or adiposity [weight: F_(3,28)_ = 1.78, p>0.05; adiposity: F_(3,28)_ = 0.45, p>0.05; data not shown]. Similar D1R and D2R numbers (Bmax) and affinities (Kd) were observed in NAc shell, NAc core and dorsal striatum between the four different feeding groups (Table 1). An 8% fructose solution was chosen for further studies because it produced robust fructose intake, corresponds to the most preferred sucrose concentration and has similar sugar content to soda and fruit juice [48].

**Table 1:** Long-term fructose bingeing did not alter D1R or D2R number.

	D1R [^3^H]-SCH23390		D2R [^3^H]-Spiperone	
	Bmax	Kd	Bmax	Kd
	(fmol/mg protein)	(pM)	(fmol/mg protein)	(pM)
**Nucleus accumbens shell**				
Chow	223±4	430±42	117±4	136±13
4% fructose	222±4	424±42	114±2	115±15
8% fructose	227±5	477±49	117±7	120±15
12% fructose	217±5	422±53	119±7	132±32
**Nucleus accumbens core**				
Chow	189±6	417±63	97±8	140±16
4% fructose	194±6	420±61	93±5	128±22
8% fructose	202±5	421±45	104±9	128±13
12% fructose	188±6	432±68	108±6	165±23
**Dorsal striatum**				
Chow	225±4	439±35	175±6	163±18
4% fructose	220±4	408±36	170±7	135±25
8% fructose	223±4	438±39	169±11	148±24
12% fructose	219±6	425±51	166±7	148±21

Mean values ± SEM, n = 8/group.

### Only the Long-term IAM Altered Neuronal Activation, Despite Both Long- and Short-term IAM Producing Bingeing

**Figure 2:**
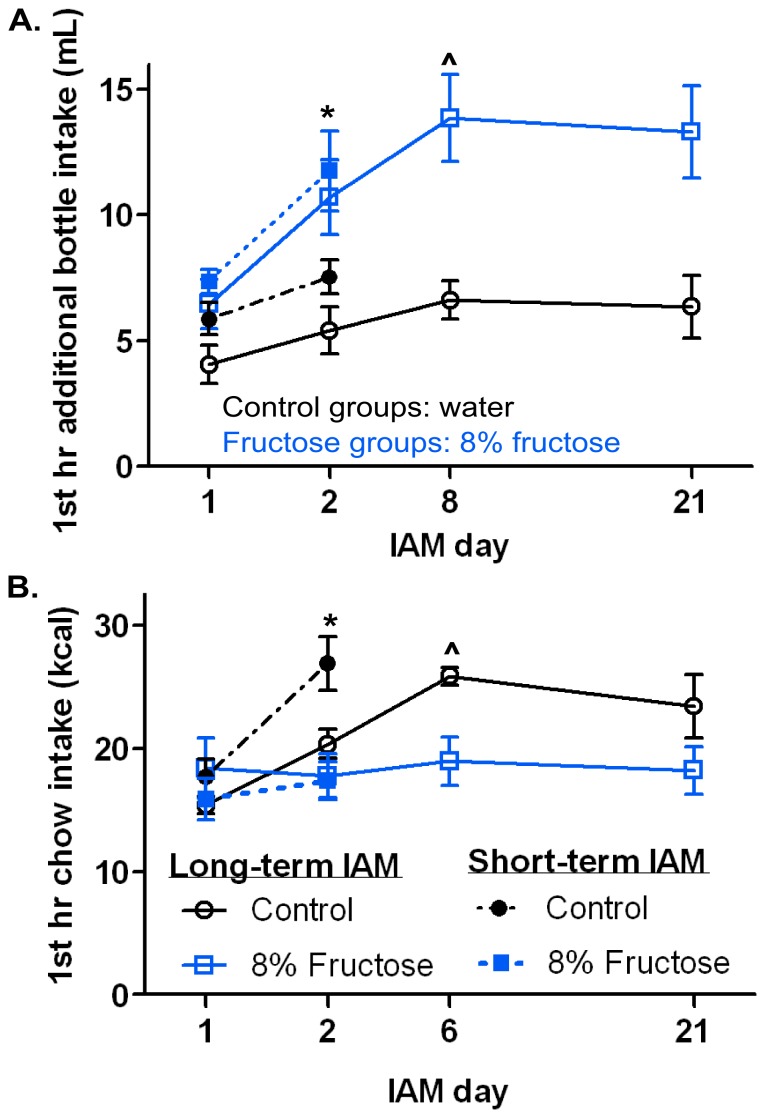
Both long- and short-term IAM produced similar levels of bingeing behavior. **A:** 1^st^ hr water (black symbols; control) or fructose (blue symbols; 8% fructose) intake on days 1 and 2 for the short-term IAM experiment (closed symbols) and on days 1, 2, 8 and 21 for the long-term IAM experiment (open symbols). **B:** 1^st^ hr chow intake on days 1 and 2 of short-term IAM and days 1, 2, 6, and 21 of long-term IAM. ∧ denotes the day at which stable fructose or chow bingeing was achieved in the long-term IAM experiment. *p<0.05 paired t-test short-term IAM only. Mean values ± SEM (n = 8/group).

We next investigated fructose-induced neuronal activation, as determined by Fos-IR, to identify the brain regions involved in long- and short-term fructose intake. To do this, rats were given either long- (21-day) or short-term (2-day) exposure to the IAM and then processed for Fos-IR following the final feeding period. A 2-day exposure was chosen to limit novelty of fructose while also remaining within the acquisition phase of the IAM. On days 1 and 2, the long- and short-term IAM cohorts had relatively similar intake within their respective feeding group (Fig. 2). Likewise, the long-term IAM produced bingeing similar to that in the 8% fructose and control groups in the initial fructose concentration-response (C-R) experiment (Figs. 2 and 1). The fructose groups from the long- and short-term IAM exhibited fructose bingeing by days 8 and 2, respectively [Long-term: F_(20,140)_ = 2.47, p<0.01; Short-term: t(6) = 2.85 p<0.05; Fig. 2A]. Similarly, chow bingeing was observed in the long- and short-term IAM control groups by days 6 and day 2, respectively [Long-term: F_(20,140)_ = 4.08, p<0.0001 Short-term: t(5) = 6.70, p<0.01; Fig. 2B]. Bingeing criteria were met by day 2 in the short-term IAM due to the statistical test being used, a paired t-test, instead of a 1-way RMANOVA used for all other IAM experiments. If a paired t-test was used to determine bingeing in the long-term cohort, the control group met criteria by day 2 [t(7) = 4.52, p<0.01] and there was a strong trend for bingeing by day 2 in the fructose group [t(7) = 2.35, p = 0.052].

**Figure 3:**
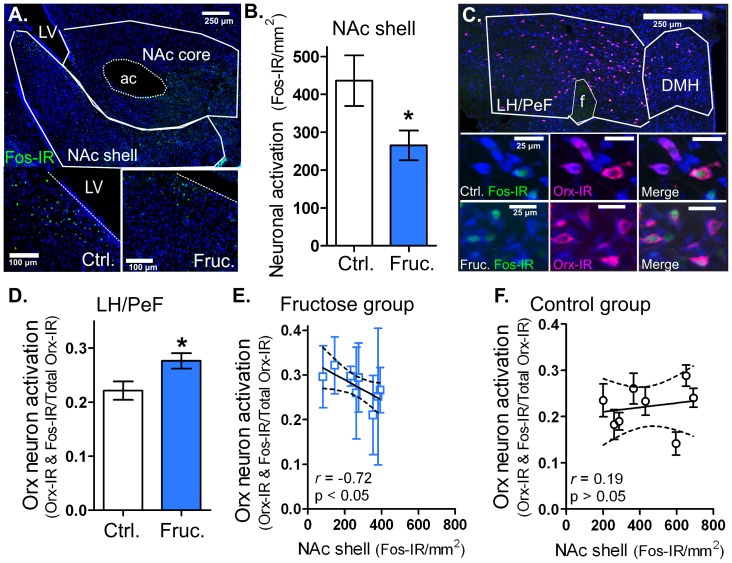
Long-term fructose bingeing reduced NAc shell neuron and enhanced LH/PeF Orx neuron activation. Immunohistochemical (IHC) staining results following long-term IAM (see Fig. 2). **A:** Anatomic outlines of NAc shell and core with enlarged representative images of IHC staining in NAc shell from control (Ctrl.) and 8% fructose (Fruc.) group animals with Fos-IR in green and Nissl stain in blue. **B:** Group results for NAc shell Fos-IR. **C:** Anatomic outlines of LH/PeF and DMH with enlarged representative images of IHC staining in Ctrl. and Fruc. group animals. The Fos-IR nuclei are in green, Orx-IR cells are in fuchsia and Nissl stain is in blue. **D:** Group results for the ratio of Orx neuron activation (Fos-IR nuclei in Orx-IR cells) in the LH/PeF. **E:** Significant negative correlation between LH/PeF Orx neuron activation and NAc shell neuron activation in the fructose IAM group. **F:** No correlation in the control IAM group. Dotted bands represent 95% confidence intervals of the linear regression. Mean values ± SEM (n = 7–8 per group) *p<0.05 unpaired t-test. Abbreviations: anterior commissure (ac), lateral ventricle (LV), fornix (f).

To assess the role of hedonic feeding centers in fructose bingeing, we measured Fos-IR in regions known to modulate sugar intake: the NAc shell and core, CeA, basolateral amygdala (BLA), and medial prefrontal cortex (mPFC). The anatomic borders defined for the NAc shell and core are shown in Fig. 3A. In the NAc shell, the long-term fructose bingeing group exhibited 40% less Fos-IR than controls [t(14) = 2.28, p<0.05; Figs. 3A and B]. NAc shell Fos-IR was similar between fructose and control groups in the short-term IAM (Table 2). Similar levels of Fos-IR were observed in both fructose and control groups in the NAc core, mPFC, CeA and BLA for both long- and short-term IAM (Table 2).

**Table 2:** Fos-IR in hypothalamic and reward-related regions.

	Long-term IAM		Short-term IAM	
	Control	Fructose	Control	Fructose
**Hypothalamic Areas**				
PVN	506±131	660±74	247±36	294±36
VMH	371±68	305±85	169±24	187±50
**Reward-Related Areas**				
NAc shell	*436±67*	265±39*	290±44	224±28
NAc core	300±26	318±34	342±25	329±25
PFC	467±74	535±65	307±24	384±30
BLA	413±45	429±42	203±31	227±28
CeA	343±30	336±40	301±34	302±40

Mean Fos-IR/mm^2^ values ± SEM, *p<0.05 control vs. fructose, unpaired t-test n = 7–8/group.

In order to explore the role of Orx neurons in long- and short-term fructose bingeing, hypothalamic sections were co-stained for Fos-IR and Orx-A immunoreactivity (Orx-IR). The anatomic borders defined for the LH/PeF and DMH are shown in Fig. 3C. In the LH/PeF, a 27% increase in Orx neuron activation (Fos-IR nuclei in Orx-IR neurons) was observed in the long-term fructose bingeing group compared to controls [t(14) = 2.47, p<0.05;Fig. 3C and D). Furthermore, there was a significant negative correlation between NAc shell activation and LH/PeF Orx neuron activation within the fructose bingeing rats [*r* = -.071, p<0.05; Fig. 3E]; this relationship was not observed in the control group [r = 0.19, p>0.05; Fig. 3F]. Following short-term IAM exposure, LH/PeF Orx neuron activation was similar between feeding groups (Table 3). Importantly, in LH/PeF, total Orx-IR neurons and non-Orx neuron activation (Fos-IR nuclei in non-Orx-IR neurons) were similar in both feeding groups following either long- or short-term IAM (Table 3). Similar Orx neuron activation was observed between groups in the DMH following long- and short-term IAM (Table 3). Interestingly, in the short-term IAM fructose bingeing animals, non-Orx neuron activation was elevated in the DMH [t(8) = 2.75, p<0.05; Table 3]. The paraventricular nucleus (PVN) and ventromedial hypothalamus (VMH) showed similar levels of Fos-IR between feeding groups for both long- and short-term IAM (Table 2).

**Table 3:** Fos-IR in Orx-IR neurons after long- or short-term IAM.

	Long-term IAM		Short-term IAM	
	Control	Fructose	Control	Fructose
**LH/PeF**				
Orx-IR & Fos-IR/mm^2^	48±7	61±11	35±3	39±6
Non-Orx-IR & Fos-IR/mm^2^	248±26	243±35	402±35	482±33
Orx-IR/mm^2^	219±23	186±20	152±11	166±20
**DMH**				
Orx-IR & Fos-IR/mm^2^	31±5	38±5	21±3	20±4
Non-Orx-IR & Fos-IR/mm^2^	158±27	177±26	*155±24*	371±78*
Orx-IR/mm^2^	166±28	146±24	112±16	102±12

Mean values ± SEM *p<0.05 control vs. fructose, unpaired t-test n = 7–8/group.

### Following Long-term IAM, Systemic Ox1R Antagonism Nonspecifically Reduced Caloric Intake

**Figure 4:**
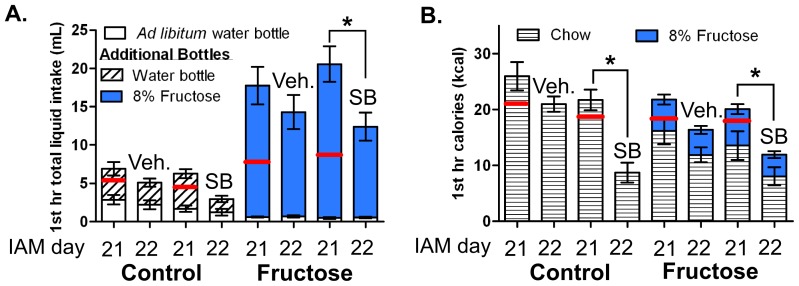
Ox1R antagonist pretreatment reduced caloric intake in both IAM feeding groups. **A:** 1^st^ hr total liquid intake for *ad libitum* water (white column) and additional bottle, containing water (diagonal striped) or 8% fructose (blue column), for control and fructose groups on day 21 of IAM (no injection) and on day 22 of IAM after a 30-min pretreatment with vehicle (2% DMSO and 10% β-cyclodextrin in sterile water; Veh.) or SB-334867 (30 mg/kg, i.p; SB) **B:** The 1^st^ hr caloric intake, expressed as chow (horizontal striped columns) and 8% fructose (in blue columns) calories. Red lines indicate day 1 intake of water or fructose in panel A and calories in panel B. Mean values ± SEM (n = 6–9/group) *p<0.05 within group 1-way RMANOVA compared to day 21.

To determine whether Ox1R signaling mediates fructose bingeing, a separate group of long-term IAM rats were pretreated with the Ox1R antagonist SB-334867 (30 mg/kg, i.p.) or vehicle, 30 min prior to food presentation on day 22 of IAM. This cohort exhibited similar bingeing on their respective foods compared to previous experiments; and all groups consumed a similar amount of 1^st^ hr calories on day 21(Fig. 4). In the fructose bingeing group, SB-334867 pretreatment caused a 40% decrease in both 1^st^ hr fructose and chow intake, as compared to the previous day’s intake, resulting in a 50% reduction in total caloric intake (Fig. 4). Similarly, the control group showed a 60% decrease in chow intake following SB-334867 pretreatment, as compared to the previous day (Fig. 4B). Overall, a 2-way ANOVA showed a main effect of drug pretreatment [F_(1,26)_ = 12.44, p<0.05], but not diet [F_(1,26)_ = 0.02, p>0.05], with no interaction [F_(1,26]_ = 1.97, p>0.05] for 1^st^ hr intake on day 22, indicating Ox1R antagonism produced similar reductions in both feeding groups. Vehicle pretreatment did not significantly alter intake of fructose, chow, or water, as compared to no injection on day 21 (Fig. 4).

### Ox1R Antagonism Reduced Fos-IR in Control, but not Fructose Bingeing, Animals

**Figure 5:**
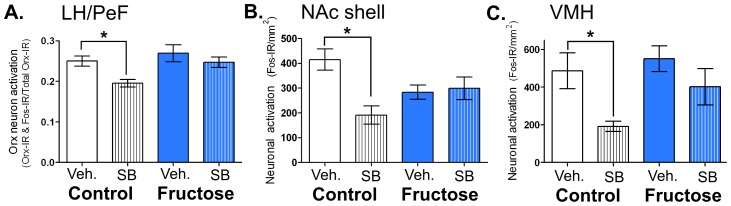
Ox1R antagonist pretreatment reduced feeding-induced neuronal activation only in the control IAM group. **A:** Orx neuron Fos-IR in LH/PeF, **B:** Fos-IR in the NAc shell, and **C:** Fos-IR in VMH following vehicle (Veh.) or SB-334867 (SB) pretreatment and 1^st^ hr bingeing on day 22 of IAM (see Fig. 4 for treatment details). Mean values ± SEM (n = 6–9 animals/group) *p<0.05 within feeding group 1-way ANOVA.

Although SB-334867 pretreatment decreased feeding in both control and fructose bingeing groups, only the control group showed reduced Fos-IR in response to drug pretreatment. Specifically, in SB-334867- compared to vehicle-pretreated control rats, Orx neuron activation was significantly reduced by 26% in the LH/PeF [F_(3,28)_ = 4.27, p<0.05] (Fig. 5A), by 54% in the NAc shell [F_(3,27)_ = 5.57, p<0.05] (Fig. 5B), and by 61% in the VMH F_(3,25)_ = 4.66, p<0.05] (Fig. 5C). In the DMH, there was a strong trend [F_(3,27)_ = 2.99, p = 0.051] for reduced Fos-IR in non-Orx neurons of SB-334867- versus vehicle-pretreated control animals (data not shown). Additionally, no differences in Fos-IR were observed between SB-334867- and vehicle-pretreatment in either feeding group in the NAc core [F_(3,25)_ = 0.35], mPFC [F_(3,23)_ = 1.28], BLA [F_ 3,22)_ = 2.31], CeA [F_(3,21)_ = 1.67], or PVN [F_(3,19)_ = 1.03] (data not shown).

## Discussion

In this study we examined if intermittent exposure to fructose produces bingeing behavior and whether fructose bingeing activates hedonic and homeostatic feeding centers. In particular, we focused on the role of Orx neurons and Ox1Rs, as they link these two feeding centers and regulate multiple aspects of food reward. Additionally, we investigated whether fructose bingeing, similar to glucose bingeing, alters DA R number in the NAc. First, we showed that 8 and 12% fructose solutions produced bingeing behavior following long-term IAM, but did not alter either D1R or D2R number. Using 8% fructose for all subsequent studies, we found that short-term IAM also produced bingeing; yet, only the long-term fructose IAM decreased NAc shell neuron activation and increased LH/PeF Orx neuron activation. Rats systemically pretreated with the Ox1R antagonist SB-334867 showed an equal reduction in caloric intake in both the fructose bingeing and control groups. Surprisingly, in the control, but not fructose, group pretreatment with SB-334867, as compared to vehicle, also reduced Fos-IR in LH/PeF Orx, NAc shell and VMH neurons. This indicates in the IAM, Ox1R signaling appears to regulate caloric- rather than palatability-driven feeding. Overall, our results suggest long-term fructose bingeing activates a hyperphagic circuit composed in part of NAc shell and LH/PeF Orx neurons. Finally, these fructose-induced changes in neuronal activation differ from those reported for glucose containing sugars, signifying individual monosaccharides may produce distinct changes in neuronal circuits involved in feeding behavior [9], [49].

Similar to other rewarding substances, we found fructose bingeing follows a typical ‘inverted U-shape’ C-R relationship. The lowest and highest concentrations, 4% and 25% fructose, failed to produce bingeing while the middle concentrations, 8% and 12% fructose, produced robust fructose bingeing (25% fructose unpublished observations). Fructose bingeing behavior was specific to the sugar solution, as chow intake remained constant over time. Rats exposed to the 4% fructose solution binged on chow, indicating that the preference for 4% fructose was not strong enough to stimulate sugar bingeing and/or occlude chow bingeing. Unlike previous IAM studies, we also observed chow bingeing in the control groups [10], [50]. Control groups in previous IAM studies also showed increased chow intake over time, but this failed to meet the statistical criteria for bingeing. We think observing bingeing behavior in both feeding groups strengthens the model by eliminating potential differences in 1^st^ hr caloric intake and feeding behavior. Overall, the IAM provides an excellent model for investigating the neuronal systems involved in fructose intake because it is well controlled and synchronizes fructose intake into large binge-like episodes.

Unlike glucose, fructose bingeing failed to alter the number of DA D1Rs or D2Rs within the NAc, suggesting long-term fructose bingeing does not chronically elevate extracellular DA levels within this region [12]. Both sweet taste and post-oral reinforcement increase extracellular DA levels in the NAc shell [51], [52]. However, recent evidence suggests that post-oral mechanisms may be more efficacious than taste in increasing extracellular DA levels [53]. Fructose reinforcement is largely taste-driven, potentially because of its inability to bind to sodium glucose transporters (SGLTs) 1 and 3, which are important in post-oral reinforcement [54]–[57]. Glucose is less sweet but stimulates robust post-oral reinforcement mechanisms potentially by binding to SGLT 1 and 3 [54], [57]–[60]. Overall, we postulate the differences in DA receptor regulation between glucose and fructose may be due to their different modes of reinforcement.

We next asked which hedonic and homeostatic feeding centers were activated by fructose bingeing following either long- or short-term IAM exposure. Even though both long- and short-term IAM fructose bingeing cohorts exhibited similar relative increases in fructose intake on day 2 versus day 1, only the long-term bingeing altered Fos-IR in the hedonic and homeostatic feeding areas investigated. This suggests that brain regions outside these areas are critical for acquiring fructose bingeing behavior, potentially the nucleus of the solitary tract (NTS) and the parabrachial nucleus, which are involved in taste [54]. Similar to other behaviors involving synaptic plasticity, a “switch” is likely to occur between the regions initiating and sustaining fructose bingeing behavior. Our Fos-IR data support the idea of NAc shell and Orx neurons being important for the expression of fructose bingeing upon repeated exposures. Future experiments are necessary to investigate the role of taste nuclei within long- and short-term fructose bingeing, as well as any “switching” mechanism.

Following long-term fructose bingeing, the rats showed less NAc shell neuron activation, which was correlated with greater LH/PeF Orx neuron activation, as compared to controls. These findings were highly specific as no other significant differences in Fos-IR were observed in the other brain regions investigated (NAc core, mPFC, CeA, BLA, PVN and VMH) or in Orx neuron activation in the DMH. Fos-IR is a unidirectional activation marker and cannot provide information about inhibition [61]. As such, the lower Fos-IR levels observed reflect decreased activation rather than inhibition. This activation pattern seems to be unique to fructose and is not seen following repeated sucrose feeding or a single exposure to intragastric glucose, both of which increase Fos-IR in the NAc but not the LH/PeF [9], [49]. Taken together, our results fit well with a feeding circuit characterized by the A.E. Kelley lab, wherein NAc shell inhibition induced by injection of either muscimol, a GABA_A_R agonist, or DNQX, an AMPA/kainate R antagonist, results in LH Orx neuron activation and marked hyperphagia of freely available food (reviewed here [62]). The VP forms a critical part of this hyperphagic circuit and provides an indirect or lateral pathway for NAc shell inhibition to activate the LH [28].

Based on the aforementioned circuit, it is possible that repeated fructose bingeing stimulates hyperphagia by sensitizing NAc shell GABAergic output neurons to opioid-induced inhibition. Repeated μ opioid receptor (MOR) stimulation via intermittent access to palatable food or intra-NAc shell infusion of DAMGO sensitizes the medium spiny neurons to inhibition, as evidenced by a leftward shift in the dose-response curve for muscimol-induced hyperphagia [63]. In addition, other bingeing models show sensitization of MORs in that the animals exhibit greater hyperphagia or hypophagia following systemic μ/κ OR agonist or antagonist treatment, respectively [64]. As a palatable food, fructose could also sensitize medium spiny neurons to inhibition, thereby reducing NAc shell GABAergic output to the LH and VP and activating Orx neurons in the LH. Our Fos-IR results support this hypothesis by showing no initial differences in NAc shell or LH/PeF Orx neuron activation, indicating repeated exposure was necessary for this effect. In addition, increased orexigenic drive could play a role in fructose bingeing. For example, NAc shell inhibition and Orx-A application decreases anorexic proopiomelancortin (POMC) neuron activity and increases orexigenic neuropeptide Y (NPY) neuron activity within the arcuate nucleus (ARC) [65]–[67]. Fructose promotes a similar orexigenic drive by decreasing POMC and increasing NPY expression within the hypothalamus, as well as by maintaining Orx neuron firing [5], [17]. Taken together, these observations provide putative mechanisms whereby fructose bingeing could inhibit NAc shell output, enhance Orx release and trigger a feeding cascade.

As noted in the Introduction, activation of Ox1Rs stimulates feeding in freely fed rats [14], [33]. Our findings suggest long-term fructose bingeing enhanced Orx-A release and thus potentially increased Ox1R signaling. In the fructose bingeing group, acute systemic pretreatment with the Ox1R antagonist SB-334867 decreased intake by 40% of both highly palatable (fructose solution) and less palatable food (chow), totaling a 50% caloric reduction. Similarly, the control group exhibited a 60% reduction in chow intake following SB-334867 pretreatment, consistent with the literature [35]. Our results indicate Ox1Rs influence feeding based on caloric content, rather than palatability in the IAM. This conclusion agrees with the literature on muscimol-induced hyperphagia, which does not occur in the presence of nonnutritive sweeteners but occurs nonselectively for high fat or high carbohydrate pellets [62]. Our study is the first to examine the effects of SB-334867 on intake of two concurrently available foods. In a different model of binge eating, the Ox1R antagonist GSK1059865, but not an Ox2R antagonist, reduced high fat, but not chow, intake. However, these rats consumed very little chow, less than 5% of total calories, making it difficult to detect decreases in chow feeding [68].

Systemic SB-334867 could have produced impairments in locomotion or drowsiness, which would have prevented the rats from feeding. However, this seems unlikely based on the literature. While SB-334867 has been shown to reduce basal locomotion, this effect disappears when palatable food is present [69], [70]. SB-334867 (30 mg/kg; i.p.) has been shown to reduce caloric intake by decreasing feeding duration and increasing rest frequency and duration, similar to another satiation signal, cholecystokinin-8, but unlike an aversive agent, lithium chloride [38], [70]. Additionally, the dose of SB-334867 used here (30 mg/kg; i.p.), but not lower doses (10 or 20 mg/kg; i.p.), attenuated sucrose self-administration with no reported off-target effects [47]. Finally, SB-334867 pretreatment alone does not alter arousal or induction of sleep but attenuates Orx-A-induced arousal [39]. Thus, the observed reductions in chow and fructose feeding were likely due to delayed satiation, rather than reduced locomotion or enhanced drowsiness.

To determine the brain regions through which systemic SB-334867 acted to attenuate feeding, rats were processed for Fos-IR following drug pretreatment and a final opportunity to binge. In the control animals, pretreatment with SB-334867, compared to vehicle, reduced Fos-IR in Orx neurons in the LH/PeF and in neurons in the NAc shell and VMH. Orx neurons in the LH express Ox1Rs; but when locally activated Ox1Rs do not regulate feeding, suggesting SB-334867 inhibition of Ox1R signaling in other regions that receive LH projections may mediate this effect [71], [72]. The NAc shell contains Ox1Rs, which reduce Orx-A-induced feeding when antagonized by local application of SB-334867 [30], [45]. The VMH is an unlikely locus for SB-334867 to reduce feeding. In the VMH Orx-A modulates glucose utilization by skeletal muscles but not feeding [4], [73]. In addition, it is possible that Ox1Rs outside of these brain regions, discussed below, could also alter chow intake. Surprisingly, in fructose bingeing rats, we did not observe any alterations in Fos-IR following SB-334867 pretreatment. Although SB-334867 has been widely used in behavioral studies, our study is the second to investigate Fos-IR following SB-334867 pretreatment. In the only other study SB-334867 pretreatment attenuated Fos-IR in the NAc shell of morphine withdrawn rats but not in controls, indicating SB-334867 is capable of reducing stimulated but not basal Fos-IR [74]. With this in mind, there are two possibilities to explain our findings in the long-term fructose bingeing animals: (1) SB-334867 antagonizes Ox1Rs in the NAc shell and LH without altering Fos-IR and/or (2) the critical loci for SB-334867 antagonism of Ox1Rs are in brain regions not studied here. In support of the first explanation, in the NAc shell Ox1Rs enhance DA release. Thus, antagonizing Ox1Rs would reduce DA levels and Fos-IR in D1R-expressing neurons while increasing Fos-IR in D2R-expressing neurons, which could result in minimal net change in Fos-IR [44]. In support of the second explanation, the ARC, NTS, VP and paraventricular nucleus of the thalamus all contain Ox1Rs with functional links to palatable food intake and could influence both fructose and chow intake in our IAM [66], [67], [75]–[77]. Feeding is a vital, complex behavior and it is likely that redundant mechanisms and several brain circuits play a role in reducing feeding following SB-334867 pretreatment in both fructose bingeing and control rats.

In summary, we found repeated, intermittent access to 8% or 12% fructose significantly modified feeding behavior and resulted in fructose bingeing. Instead of activating the NAc shell, similar to reports for glucose containing sugars, fructose bingeing reduced NAc shell and increased LH/PeF Orx neuron activation, which mirrors a well-established feeding circuit and points to the involvement of both hedonic and homeostatic mechanisms. Additionally, we were the first to show Ox1R antagonism equally attenuated intake of concurrently available high and low palatability foods. These results suggest that in the IAM Ox1Rs influence calorically-based, rather than palatability-based, feeding. Overall, the enhanced Orx signaling produced by fructose bingeing could create a positive feedback cycle promoting continued fructose bingeing behavior and may generalize to overeating beyond homeostatic needs. In addition, enhanced Orx signaling would have profound effects on arousal, energy expenditure, attention to food-associated cues, and motivation, all of which could influence food choices and consumption. More broadly, our results and the literature suggest fructose and glucose may stimulate feeding behavior through distinct brain circuits. Further research is needed to elucidate the brain mechanisms that promote bingeing of individual monosaccharaides, as well as complex sugar mixtures.
